# Spin Purification in Full-CI Quantum Monte Carlo via a First-Order Penalty Approach

**DOI:** 10.1021/acs.jpca.2c01338

**Published:** 2022-03-17

**Authors:** Oskar Weser, Niklas Liebermann, Daniel Kats, Ali Alavi, Giovanni Li Manni

**Affiliations:** † Max-Planck-Institute for Solid State Research, 70569 Stuttgart, Germany; ‡ Department of Chemistry, University of Cambridge, Lensfield Road, Cambridge CB2 1EW, U.K.

## Abstract

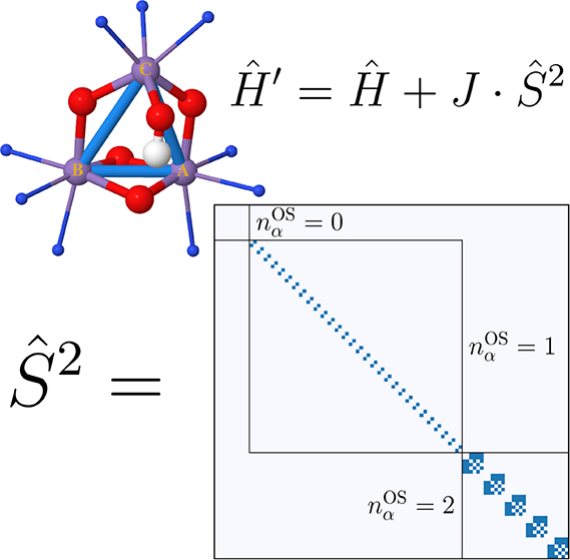

In this article, we demonstrate that a first-order spin penalty scheme can be efficiently applied to the Slater determinant based Full-CI Quantum Monte Carlo (FCIQMC) algorithm, as a practical route toward spin purification. Two crucial applications are presented to demonstrate the validity and robustness of this scheme: the ^1^Δ_*g*_ ← ^3^Σ_*g*_ vertical excitation in O_2_ and key spin gaps in a [Mn_3_^(IV)^O_4_] cluster. In the absence of a robust spin adaptation/purification technique, both applications would be unattainable by Slater determinant based ground state methods, with any starting wave function collapsing into the higher-spin ground state during the optimization. This strategy can be coupled to other algorithms that use the Slater determinant based FCIQMC algorithm as configuration interaction eigensolver, including the Stochastic Generalized Active Space, the similarity-transformed FCIQMC, the tailored-CC, and second-order perturbation theory approaches. Moreover, in contrast to the GUGA-FCIQMC technique, this strategy features both spin projection and total spin adaptation, making it appealing when solving anisotropic Hamiltonians. It also provides spin-resolved reduced density matrices, important for the investigation of spin-dependent properties in polynuclear transition metal clusters, such as the hyperfine-coupling constants.

## 1 Introduction

Strongly open-shell molecules present a number of challenges to quantum chemical methods, arising from the large number of nearly degenerate states with different total spin quantum number, *S*, which exist in such systems and are in general hard to resolve. In these systems, spin contamination is a major problem for an accurate description of their electronic spectrum. Such systems usually exhibit a strong *multireference* character, with numerous dominant electronic configurations featuring similar weights in the configuration interaction (CI) expansion. Furthermore, when a high-spin state is the ground-state, states of the same symmetry but with lower spin are impossible to obtain with ground state projective techniques. For these reasons, there has been much interest in recent years in developing spin-adapted approaches, which work in Hilbert spaces of *configuration state functions* (CSFs), rather than Slater determinants (SDs).^1−16^ In these approaches, *Ŝ*^2^ symmetry is explicitly enforced, ensuring zero spin contamination, and enabling the targeting of any desired spin state. The Graphical Unitary group approach (GUGA)^17−26^ is one such example of a fully spin-adapted approach, which was implemented within the stochastic full-CI quantum Monte Carlo (FCIQMC)^9,27−30^ and the Stochastic-CASSCF^10,31,32^ frameworks. Recently, we have discovered a strategy within GUGA that allows an unprecedented reduction of the multireference character (compression)^32−35^ of ground- and excited-state wave functions and the unique possibility to perform state-specific optimizations of ground- and excited-state wave functions.^34,35^ These properties arise from a unique block-diagonal structure of the GUGA Hamiltonian matrix, even within the same spin-symmetry sector, that follows chemically/physically motivated molecular orbital transformations.^34^ This strategy has been applied to exchange-coupled polynuclear transition metal clusters with a large number of localized open-shell orbitals^32−35^ and to one-dimensional Heisenberg and Hubbard model Hamiltonians.^36^ In the latter cases, a connection with the concept of *alternancy symmetry* can be envisioned.^37,38^ Other sparse FCI solvers^2,39−50^ could also benefit from the enhanced sparsity of the Hamiltonian and wave functions that follow the above-mentioned strategy.

However, such sophisticated approaches to spin adaptation incur a number of complications related to their increased algorithmic complexity, including matrix element calculation and excitation generation process.^9,32^ In addition it is possible^51−53^ but complicated to describe spin projection properties in a spin adapted basis, which is, e. g., necessary for anisotropic Hamiltonians or the calculation of spin polarization. Furthermore, in systems with a more delocalized character (i. e., covalency), the aforementioned compression advantages of the GUGA method are less evident. For these reasons, it is highly desirable to have a Slater determinant based approach to spin adaptation, which is possible via *spin-purification* concepts.

For cases with an even number of unpaired electrons, such as the oxygen dimer discussed later, it is also possible to place constraints on the spin by applying time-reversal symmetry and by working with pairs of spin-coupled functions.^54^ This reduces the size of the Hilbert space by a factor of 2, while reducing any spin contamination, as in the reduced space either all even or odd spin states can be excluded. However, this strategy cannot separate singlet from quintet, nor can it operate in cases with an odd number of unpaired electrons.

The aim of the present article is to introduce one such method, based on a simple first-order spin-penalty approach, within the context of Slater determinant based FCIQMC. Spin purification techniques, including the first-order spin penalty approach, have recently been discussed in details by Levine and co-workers^55^ and have already been utilized in the context of renormalization approaches.^56,57^ We build on the existing literature by explaining the origin for a range of optimal spin penalty parameters.

The remainder of the article is organized as follows. In the next section, we explain the theory behind the first-order spin-penalty method and explain the origin of the optimal spin penalty parameter. Section 3 contains two applications, the oxygen dimer and a trinuclear manganese cluster, to showcase the general applicability of this method in FCIQMC. Section 4 contains a summary and conclusion of our results. The Appendix (Section 5) contains detailed derivations of the convergence speed for different choices of the spin penalty parameter.

## 2 Theory

### 2.1 First-Order Spin Penalty Method

We write the total spin operator, *Ŝ*^2^, in terms of the spin projection, *Ŝ*_*z*_, and the ladder operators, *Ŝ*_+_ (raising) and *Ŝ*_–_ (lowering) (see also ref (58)), namely
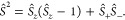
1

**Figure 1:**
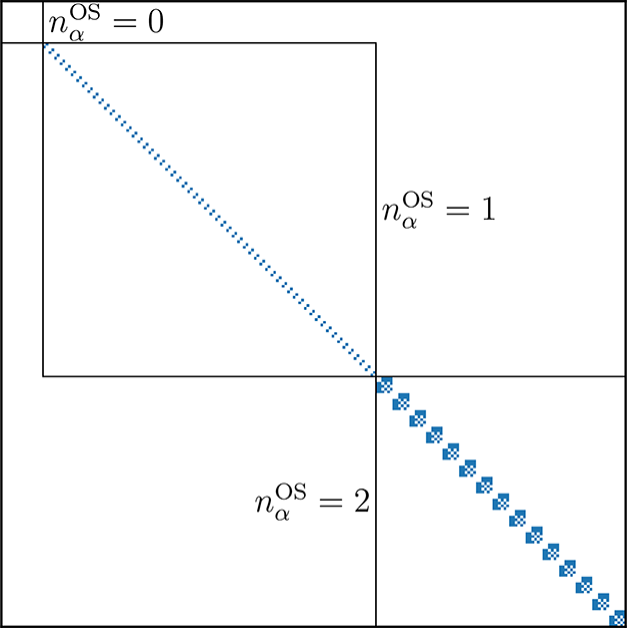
Block-diagonal structure of the *Ŝ*^2^ matrix in the SD basis of an (8,6) active space with *S*_*z*_ = 0, corresponding to a minimum active space for the singlet state of oxygen. White denotes zero, while blue denotes ±1 entries. The diagonal is omitted.

Given two SDs, |*D*_*i*_⟩ and |*D*_*j*_⟩, the expression for ⟨*D*_*i*_|*Ŝ*^2^|*D*_*j*_⟩ is then given by
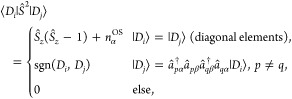
2where *n*_α_^OS^ is the number of unpaired (open-shell, OS) α electrons. The off-diagonal elements are nonzero only for exchange excitations and are equal to sgn(*D*_*i*_, *D*_*j*_) = ±1, where the sign is given by the product of Fermionic phase factors.^58^ Since exchange excitations require the same orbital configuration in both determinants, the *Ŝ*^2^ matrix features an interesting block-diagonal structure. Larger blocks are characterized by a common *n*_α_^OS^ value, while the sub-blocks are characterized by a common occupation number vector. This block-diagonal and sparse structure (see Figure 1) is particularly well suited for FCIQMC.

In the first-order spin penalty approach, a modified Hamiltonian
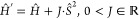
3is utilized. If *J* is chosen such that the low-spin state becomes the lowest state in the modified *Ĥ*^′^ Hamiltonian, ground state methods, including FCIQMC, will converge to that state. The on-the-fly evaluation of modified Hamiltonian matrix elements does not require additional memory, and has negligible runtime costs for the evaluation of the *Ŝ*^2^ correction. Since *Ĥ* and *Ŝ*^2^ commute, the eigenstates of *Ĥ*^′^ are still eigenstates of *Ĥ* and the eigenvalues of *Ĥ* can be directly calculated from the corresponding eigenvalues of *Ĥ*^′^ by subtracting *J*·*S*(*S* + 1). Note that this subtraction can be performed in a well-defined manner only for converged eigensolutions. For unconverged, intermediate results, for example along FCIQMC dynamics and before stationary conditions are reached, it is necessary to evaluate directly the original Hamiltonian *Ĥ*. In the present work we calculate the latter as an expectation value from the stochastically sampled one- and two-body reduced density matrices (RDMs).

### 2.2 Range of Optimal *J* Values

In the following, we discuss an optimal choice of the *J* value. For the unique *J* that makes all high-spin states energetically above or degenerate to the targeted spin state (*first flipping point*), a spin-symmetry-broken wave function is to be expected, which is an arbitrary admixture of the degenerate spin states in the modified Hamiltonian. This *flipping point* satisfies the following relation
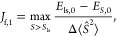
4where *E*_ls,0_ and *E*_*S*,0_ are the nonpenalized ground-state energies of the targeted low-spin (ls) and the higher spin (*S*) state and Δ⟨*Ŝ*^2^⟩ = *S*(*S* + 1) – *S*_ls_(*S*_ls_ + 1) corresponds to the difference in their spin expectation values. For *J* values larger than the first flipping point the desired low-spin state is obtained in the long-time limit of the FCIQMC dynamics. However, the speed of convergence and stability of the imaginary-time propagation in FCIQMC depends on how far *J* is from the first flipping point. At first glance, one could expect that, above the first flipping point, higher *J* values only improve the speed of convergence, because they increase the energy of the high-spin states, so an imaginary-time propagation with the modified Hamiltonian exp(−τ(*Ĥ*^′^ – *E*_0_)) projects them out faster. But, as we (*vide infra*) and Levine et al.^55^ observed, there exist *J* values beyond which the convergence deteriorates. Intuitively this can be understood, because for increasingly larger *J* values the modified Hamiltonian can be interpreted as mainly a *Ŝ*^2^ operator, corrected by a small perturbation represented by *Ĥ*. Diagonalization of the pure *Ŝ*^2^ operator results in numerous degenerate eigenstates with equivalent spin eigenvalues (not energies), the lowest being *S*(*S* + 1) = 0 or , for even and odd numbers of electrons, respectively. This degeneracy is in part lifted by the Hamiltonian *Ĥ*. However, for very large *J* values, the *Ĥ* correction becomes relatively small compared to *J*·*Ŝ*^2^, and projecting out the high-energy states of same spin becomes harder.

**Figure 2:**
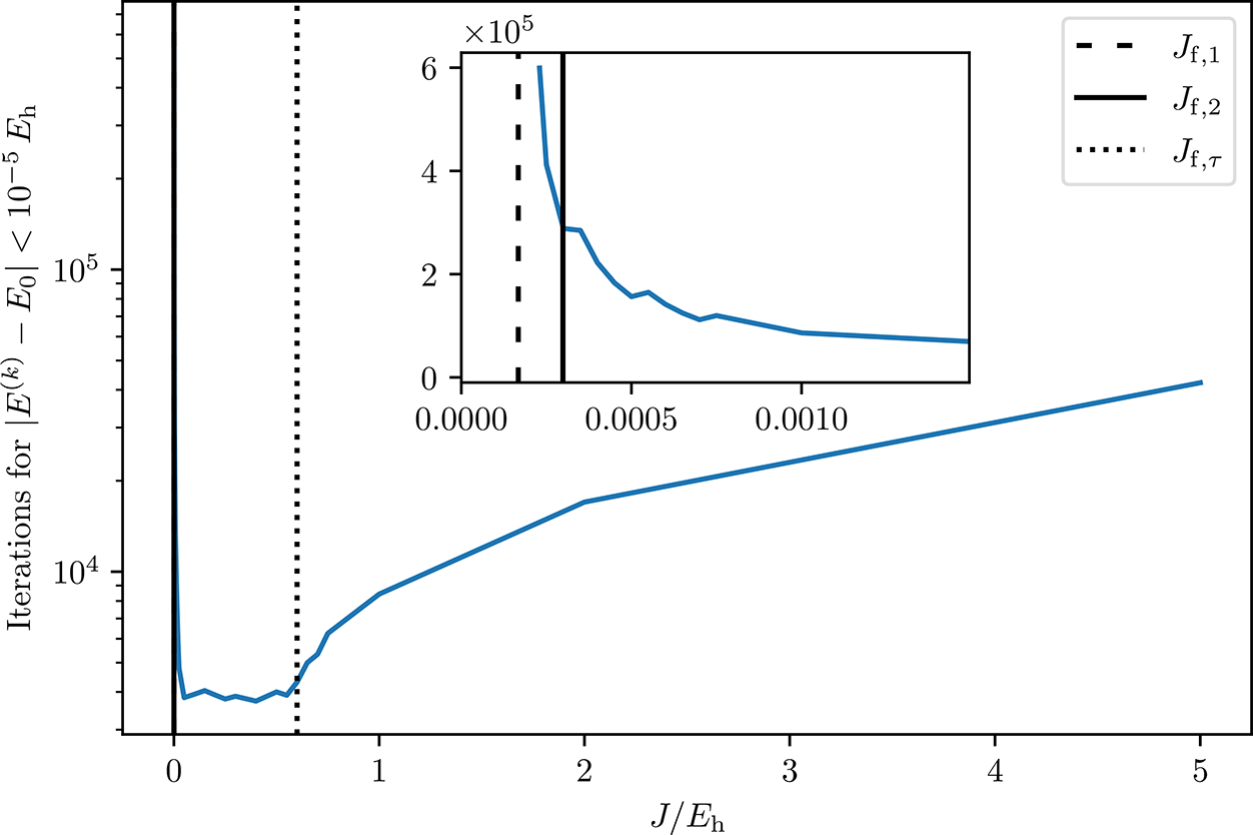
Number of iterations required to achieve convergence up to 1 × 10^–5^*E*_h_ using *deterministic* imaginary-time propagation for the Γ^(1/2)^ state of the manganese cluster in an (9,9) active space. *J*_f,1_ was calculated using eq 4, *J*_f,2_ was calculated using eq 5, and *J*_f,τ_ was calculated using the spread (*E*_max_^′^ – *E*_0_^′^), which was approximated from the spread of diagonal values of *Ĥ*^′^.

If we look at qantitative estimates for the speed of convergence (the detailed derivations are provided in the Appendix (Section 5)), an interval of *J* values exists inside which the speed of convergence is nearly constant and optimal (see Figure 2). The lower bound of this optimal range can be estimated by the *second flipping point*
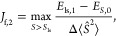
5where *E*_ls,1_ is the energy of the first low-spin excited state of desired spin. The speed of convergence increases proportionally to the energy separation between the lowest and the second-lowest state. For *J*_f,1_ < *J* < *J*_f,2_, we have *E*_ls,0_^′^ < *E*_hs,0_^′^ < *E*_ls,1_^′^, and the spread between lowest and second to lowest energy state increases with *J*. For *J* > *J*_f,2_, we have *E*_ls,0_^′^ < *E*_ls,1_^′^ < *E*_hs,0_^′^, and the energy separation between lowest and second-lowest energy state is unaffected by *J* (under the assumption that they have the same spin multiplicity). Thus, for *J* > *J*_f,2_, but still below the upper bound discussed in the following, the convergence is nearly independent of *J* (plateau in the speed of convergence, Figure 2).

For optimization techniques based on the imaginary-time Schrödinger equation, such as FCIQMC, the upper bound of the optimal range of *J* is given by the *τ-flipping point*, *J*_f,τ_, which denotes the point where the maximum time step starts to be dominated by a 1/*J* proportionality. In the case of *deterministic* imaginary-time propagations we have Δτ = (*E*_max_^′^ – *E*_0_^′^)^−1^. If we assume that the highest energy state in *Ĥ*^′^ (for increasing *J* > *J*_f,1_) and the energetically lowest spin-state have the same spin multiplicity, their energy difference will not be affected by *J* for a large range of *J*. For typical full-valence active space calculations this is a well-founded assumption. For *J* > *J*_f,τ_, the spread of *Ĥ*^′^ becomes increasingly dominated by the spread of spin expectation values, *E*_max_^′^ – *E*_0_^′^ ≈ *J*Δ⟨*Ŝ*⟩_max_, hence Δτ ≈ (*J*Δ⟨*Ŝ*⟩_max_)^−1^. Thus, larger *J* values lead to smaller optimal Δτ values, with a consequent reduction of the speed of convergence.

In the case of *stochastic* imaginary-time propagation, as in FCIQMC, the *J*_f,τ_ is more complicated to find. The time step has to be chosen differently compared to the deterministic case to achieve stable dynamics. The conventional^9,27^ choice for Δτ in a SD basis is
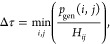
6which not only depends on the spectrum of *Ĥ* but also on the excitation generator in use and the determinant pairs *i*, *j* where spawns actually happened during a calculation. Note the implicit assumption, that the stochastic Δτ from eq 6 is smaller than the deterministically chosen Δτ, that depends purely on the spread of the spectrum of *Ĥ*. As in the deterministic case, there will be a *J*_f,τ_ from which the time step purely follows a (1/*J*) dependency. If a weighted excitation generator is in use, the *p*_gen_ for exchange excitations will increase with *J* as do the *H*_*i*,*j*_ and depending on the dynamics there will be different pairs *i*, *j* fullfilling the minimum of eq 6. Hence unlike the deterministic case there might be changes of Δτ already before reaching *J*_f,τ_. We also observe that for too large *J* the stochastic noise of the Monte Carlo simulation increases.

In summary, the convergence improves with increasing *J* for *J*_f,1_ < *J* < *J*_f,2_. Also, there exists a *J*_f,τ_ after which a 1/*J* dependency of the time step follows, negatively affecting the speed of convergence. Between *J*_f,2_ and *J*_f,τ_ there is a plateau of nearly optimal *J* values. We would like to point out (see Figure 2) that the convergence deteriorates slower for *J* > *J*_f,τ_, than for *J* < *J*_f,2_; i. e., it is better to select a bit too high *J* values than too small ones. In addition a too large *J* > *J*_f,τ_ affects the convergence rate, while a too small *J* < *J*_f,1_ leads to spin contamination. Therefore, it is advisable to choose *J* values inside the range but closer to *J*_f,τ_.

In practical applications, it is difficult to calculate the lower bound *J*_f,2_ because it requires knowledge of the spin-state energetics, which are exactly the purpose of the calculation. However, the *J*_f,τ_ can be estimated from monitoring the time step and stochastic noise during FCIQMC *training runs* (using low walker populations) for different *J* values. The first flipping point *J*_f,1_ can be found by monitoring the spin expectation value for different *J*; if it converges to a high-spin state, the *J* is too small. We assume *J*_f,τ_ – *J*_f,2_ ≈ *J*_f,τ_ – *J*_f,1_ and select a trial *J* between *J*_f,1_ and *J*_f,τ_. In the case of *stochastic* imaginary-time propagation, it is generally advised to also monitor the stochastic noise and reduce *J* accordingly.

## 3 Application

The robustness of the spin penalty method in SD-based FCIQMC has been explored in two crucial test-case applications. We investigated the vertical ^1^Δ_*g*_ ← ^3^Σ_*g*_ transition in the O_2_ molecule using a full-CI expansion in a double-ζ quality basis set, and the vertical Γ^(1/2)^ ← Γ^(3/2)^ (and Γ^(9/2)^) transition in a [Mn_3_^(IV)^O_4_] trinuclear cluster. In both cases, the ground state is the higher spin-state.

### 3.1 Oxygen Dimer

We used a distance of 1.203 Å, and correlated 16 electrons in the 28 orbitals of an ANO-RCC-VDZP basis.^59,60^ The Full-CI calculations were performed on the basis of the state-specific CASSCF(8,6) orbitals. A spin-pure calculation using GUGA-FCIQMC served as reference. The *J* parameter was set to 0.12 *E*_h_.

**Figure 3:**
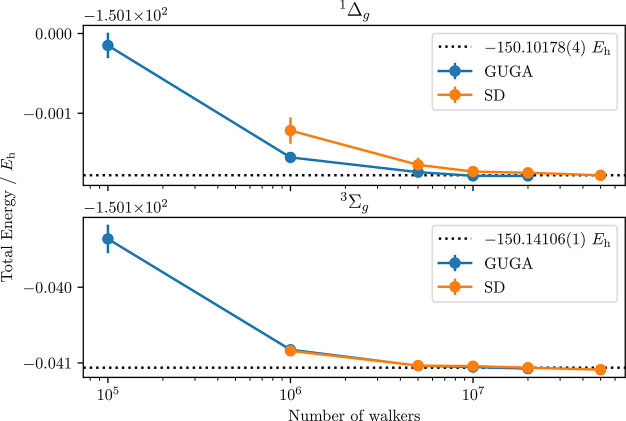
Total energy calculated from RDMs for the ^1^Δ_*g*_ (upper plot) and the ^3^Σ_*g*_ (lower plot) spin states of the oxygen molecule.

The ⟨*Ĥ*⟩ expectation value calculated from RDMs is shown in Figure 3. The triplet converges faster than the singlet, and its total energy nearly matches the energy of the GUGA reference calculation for the same walker number. Generally, convergence with respect to the number of walkers in initiator-FCIQMC is mainly influenced by the compactness of the respective wave function. The triplet calculation started from a SD with |*M*_*S*_| = 1, which is also the spin-pure configuration that dominates the FCI wave function for this electronic state. On the contrary, in the case of the singlet spin state, a multideterminantal wave function is required to correctly describe the spin-pure reference space; therefore, the calculation converges slower with respect to the walker number than the GUGA-based one. However, the wall clock time to achieve the same quality of convergence is roughly comparable, since SD-based FCIQMC is generally faster for a given walker number, as discussed in the literature.^9,33^ For all walker populations, the spin expectation values have been used to confirm convergence to the correct spin state and monitor spin contamination. The deviation was larger for the singlet whose mean spin expectation value was 1.30 × 10^–5^, compared to the theoretical 0.

### 3.2 Manganese Cluster

**Figure 4:**
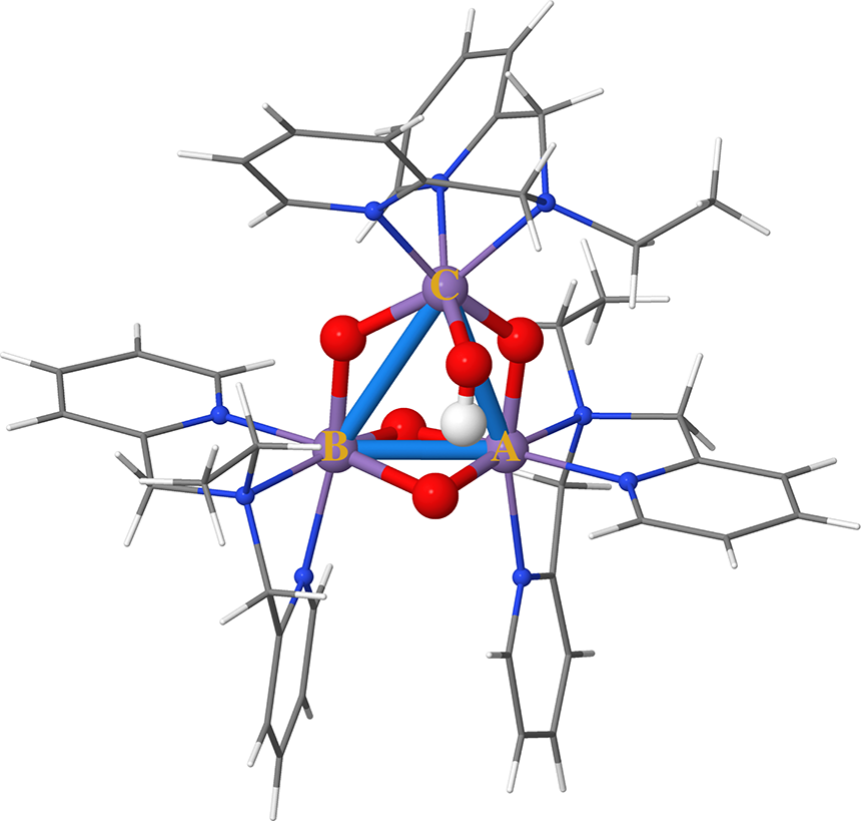
Structure of the [Mn_3_O_4_] trinuclear model system extracted from ref (61). A, B, and C labels identify the Mn(IV) magnetic centers. Oxygen, nitrogen, carbon, and hydrogen atoms are labeled in red, blue, gray, and white, respectively.

Two active spaces have been defined to test the spin penalty approach on the [Mn_3_^(IV)^O_4_] trinuclear cluster (Figure 4). A small CAS(9,9) is utilized to directly compare to the fully deterministic GUGA-based spin gap.^62,63^ A larger CAS(55,38) has been employed to demonstrate the numerical stability of the method in more realistic scenarios. The CAS(9,9) consists of the nine singly occupied *t*_2*g*_ orbitals on the three magnetic centers. The large active space consists of the 15 3d orbitals and their nine electrons, the 12 doubly occupied 2p orbitals of the bridging oxygen atoms, the 10 doubly occupied peripheral lone-pair orbitals pointing at the metal sites, and two doubly occupied orbitals of the –OH group, of σ and π character. A similar active space has been previously chosen for a similar [Mn_3_O_4_] cluster.^35,64^

Through experimental investigation, Armstrong showed that the ground state of this system is a Γ^(3/2)^ spin state.^61^ The too small CASSCF(9,9) erroneously predicts a Γ^(9/2)^ ground state. Nonetheless, this small model calculation represents an interesting test case to explore the applicability of the spin penalty strategy. The larger CAS(55,38) describes qualitatively well the spin-state ordering, with a  ground state and a  state at slightly higher energy, in line with Armstrong’s findings.

**Figure 5:**
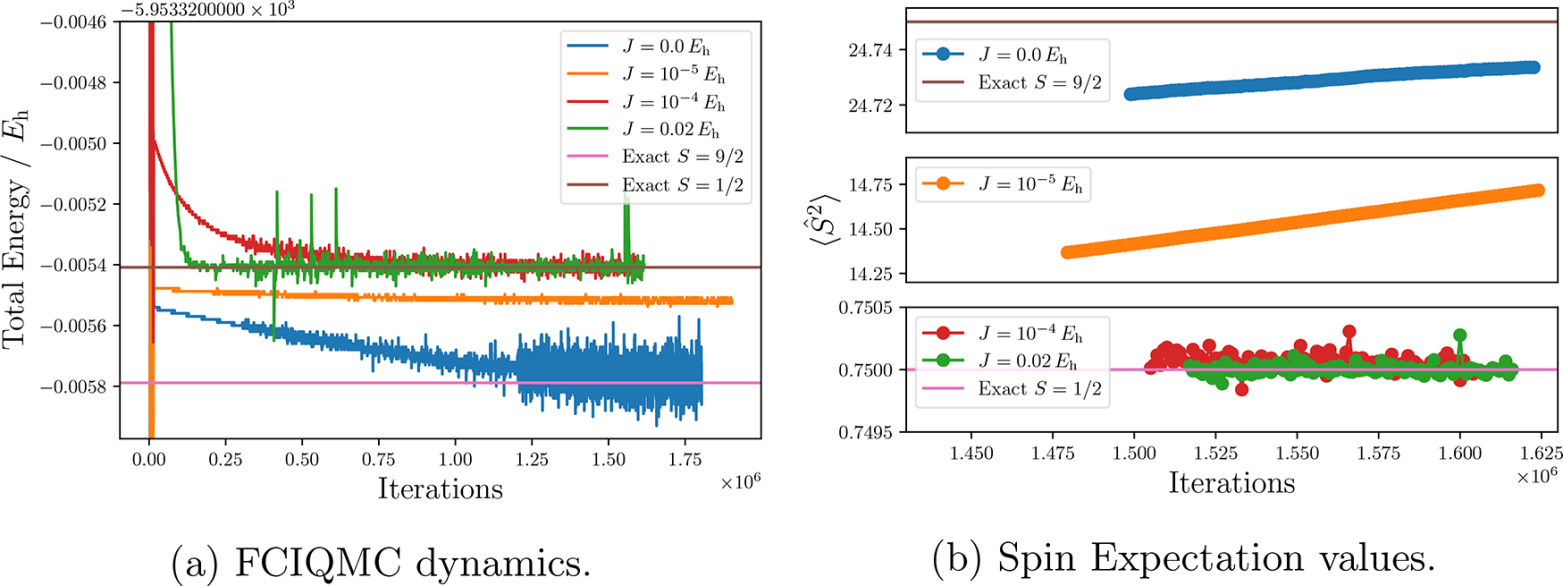
(a) FCIQMC dynamics varying the amount of spin penalty. The projected energy shifted by the *J*·*S*(*S* + 1) value is reported, where *S* is the *expected* spin value. (b) Spin expectation values calculated from RDMs are shown. All simulations used 5 × 10^4^ walkers.

In Figure 5, we show the convergence behavior of the FCIQMC dynamics for different *J* applied to the CAS(9,9) wave function. For FCIQMC dynamics with *J* = 0 *E*_h_ or too small spin penalties (J = 1 × 10^–5^*E*_h_) the flipping point is not reached, and the FCIQMC dynamics converges to the high-spin state , which is the ground state for the small CAS(9,9) model active space. For *J* values above the flipping point, the low-spin state wave function is obtained. These results are confirmed by the total spin expectation value (Figure 5b). Speed of convergence increases for larger penalty values, and a large range of *J* values (1 × 10^–4^*E*_h_ ≤ *J* ≤ 2 × 10^–2^*E*_h_) exists that provides stable and fast converging FCIQMC dynamics. Too large *J* values (>1 × 10^–1^*E*_h_) lead to convergence problems, which is in line with *J*_f,τ_ = 0.1 *E*_h_.

**Figure 6:**
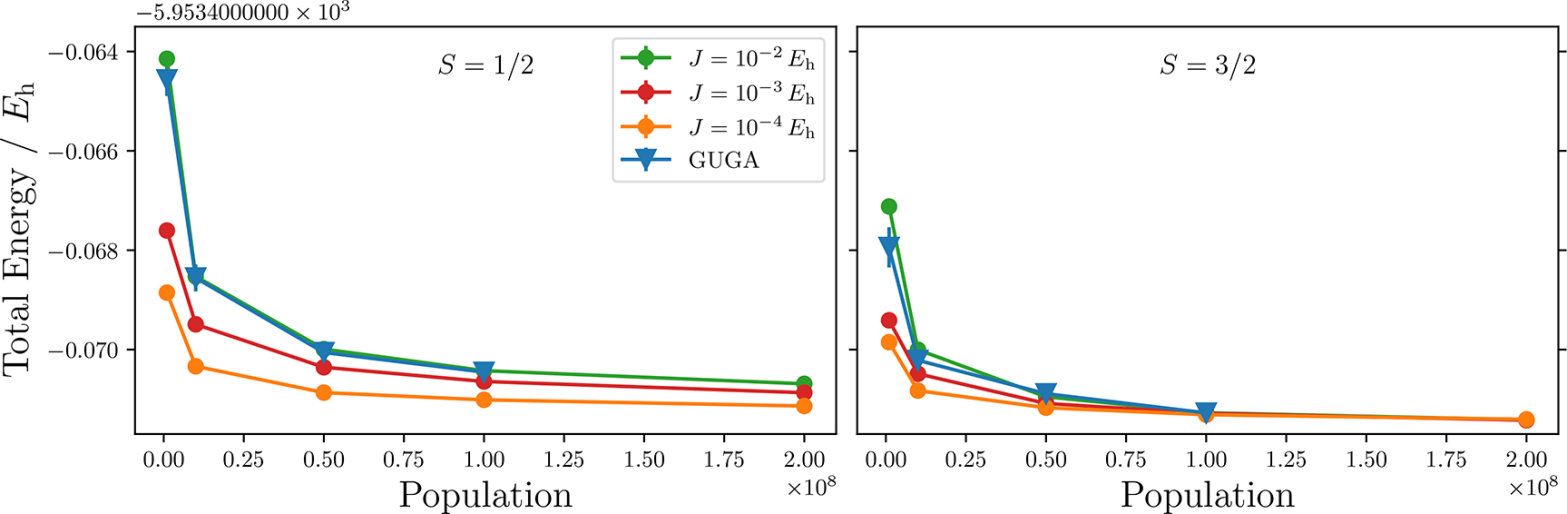
CAS(55,38) total energies, obtained as expectation values from one- and two-body RDMs, for the  (left) and  (right) spin states as a function of the walker population. The lower energies for smaller *J* values are not to be interpreted as a faster convergence with respect to walker number. They are a consequence of the admixing of the targeted spin state with higher spin states (details in the main text).

For the CAS(55,38) model active space the competing doublet  and quartet  spin state wave functions have been optimized. GUGA-FCIQMC has been utilized as a reference. Three choices of *J* were used that permitted the characterization of the doublet spin state, namely *J* = 1 × 10^–2^*E*_h_, 1 × 10^–3^*E*_h_, and 1 × 10^–4^*E*_h_. Figure 6 shows the energetics for the  and  states, as a function of the walker population. We notice that all dynamics are stable and fast converging. The choice of the large parameter, *J* = 1 × 10^–2^*E*_h_, results in a nearly exact matching of the spin-purified total energy with the one obtained from the spin-adapted GUGA-FCIQMC approach, at the same walker population.

**Figure 7:**
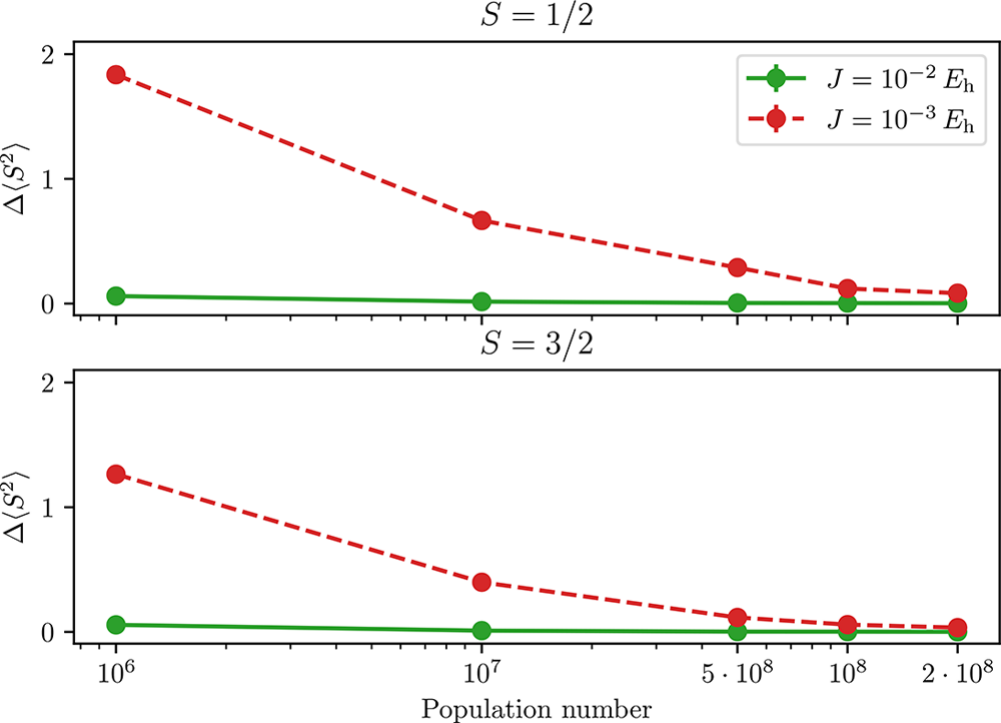
Spin contamination (Δ⟨*Ŝ*^2^⟩ = ⟨*Ŝ*^2^⟩ – *S*(*S* + 1)) in the CAS(55,38) for different *J* and population numbers. The spin ⟨*Ŝ*^2^⟩ was calculated from RDMs. While the larger *J* = 1 × 10^–2^*E*_h_ value provides dynamics with nearly exact spin expectation values for any chosen walker population, the smaller *J* = 1 × 10^–3^*E*_h_ calculations converge to the correct spin expectation value more slowly and only for larger populations.

Lower *J* values result in lower total energies for low walker populations. The lower energies for smaller *J* values are not to be interpreted as a faster convergence of the spin penalty approach for lower *J*. Instead, considering that the spin expectation value for the smaller *J* = 1 × 10^–3^*E*_h_ is higher than the expected value (Figure 7) we are brought to the conclusion that the unconverged wave function (low population) is in a broken-symmetry state, that results from the mixture of the target spin state (Γ^(1/2)^) and the higher spin states (for example Γ^(3/2)^). Admixing the higher spin states artificially lowers the total energy. For larger walker populations and for larger *J*, the spin expectation value gets closer to the targeted value, eliminating any spin contamination from the optimized wave function.

It is worth noting that the 38 active orbitals have been localized and reordered to reach maximum compression of the GUGA wave function (see refs (33−35) for details). In Figure 11 of ref (34), we have shown that GUGA-FCIQMC converges faster than the Slater determinant FCIQMC counterpart, when using a tailored (in this case localized) MO basis. Thus, we expect that the chosen one-electron basis utilized for the CAS(55,38) calculation in general favors the GUGA-FCIQMC approach. However, we observe very similar convergence of the GUGA and the Slater determinant based spin-penalty approaches (for *J* = 1 × 10^–2^*E*_h_). Moreover, it is interesting to notice that for an equivalent wall-clock time, the spin penalty approach can be run at higher walker population (2 × 10^8^ walkers in the spin penalty method versus 1 × 10^8^ walkers in GUGA) and reaches a lower total energy. These results suggest an overall better performance of the spin-purification approach. However, the GUGA strategy has two crucial advantages that we have documented in recent works:^32−35^ (a) within GUGA the space of the leading electronic configurations (CSFs) can be greatly reduced and directly connected to physical concepts, and (b) the GUGA CI Hamiltonian matrix has a unique *quasi-block-diagonal* structure, allowing for unprecedented *state-specific* optimizations of ground and/or excited states.

As a final remark, we observe that the spin-penalty strategy enables the combined *S*- and *M*_*S*_-adaptation. This scheme is thus more flexible than the GUGA *S*-adaptation, and allows for the treatment of anisotropic Hamiltonians. One trades simplicity and universality for lower dimensionality when going from the *M*_*S*_-adapted space to the *S*-adapted one. Moreover, while stochastically sampled higher-order density matrices are already available within the SD-based FCIQMC approach, allowing for multireference second order perturbation theory (PT2) methods,^65,66^ GUGA-FCIQMC three- and four-body density matrices are not available, preventing for the moment GUGA-based PT2 strategies. Additionally, it is possible to envision spin-pure similarity-transformed FCIQMC calculations based on transcorrelated methods^67−74^ using the current spin penalty approach, while technical difficulties exist within the GUGA scheme, because of the presence of three-body interactions in the transcorrelated Hamiltonian.

## 4 Conclusion

In conclusion, we have demonstrated that spin purification based on a first-order spin penalty can be efficiently applied to the Slater-determinant based FCIQMC algorithm. We have also explained the origin of an optimal range of *J* values and that too large *J* parameters are to be avoided as they result in smaller time steps, deteriorating convergence. The method was successfully applied to calculate the ^1^Δ_*g*_ ←^3^Σ_*g*_ transition in the O_2_ dimer and the Γ^(1/2)^ ←Γ^(3/2)^ (and Γ^(9/2)^) transition in a [Mn_3_^(IV)^O_4_] trinuclear cluster model. The range of applicability of the spin penalty FCIQMC approach is very broad, including the coupling of large active space Stochastic-CASSCF and Stochastic-GASSCF wave functions to methods capable of recovering dynamic correlation outside the active space, such as MC-PDFT,^75,76^ and tailored-CC,^100^ and crucially methods that require high-order interactions (for example in the form of three- and four-body RDMs) such as PT2 and similarity-transformed techniques. A large range of chemical systems and models for solid state materials can be investigated, including ferromagnetic superconductors of practical interest, such as UGe_2_^77^ and URhGe.^78^ The method can also be extended to model Hamiltonians, such as the Hubbard model, often used to investigate spin interactions in strongly correlated materials. By this approach we are able to tackle anisotropic Hamiltonians and, as *spin-resolved* density matrices are available, spin-dependent properties, such as the hyperfine coupling tensors (pivotal in characterizing spin interactions in polynuclear transition metal clusters), are within reach. These aspects will be the subject of future work.

## Figures and Tables

**Figure 8:**
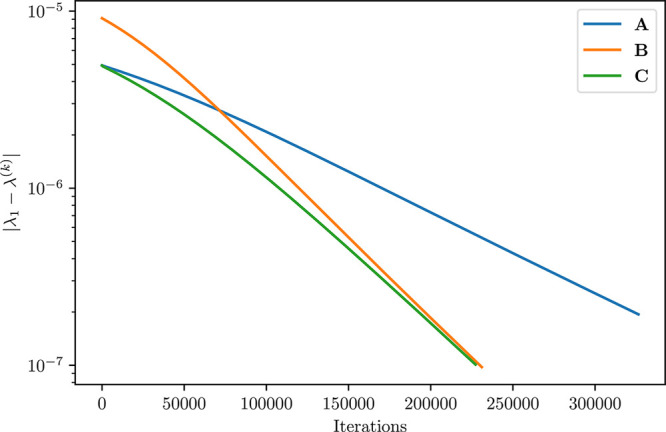
Error against the iteration number for the power method applied to matrices **A**, **B**, and **C** from eq 14.

**Figure 9:**
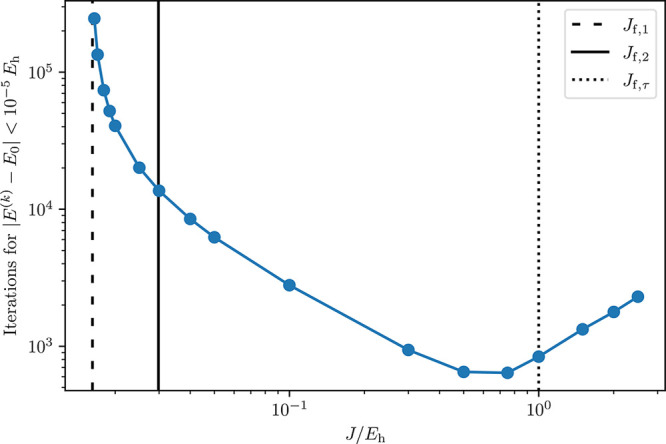
Number of iterations required to achieve convergence up to 1 × 10^–5^*E*_h_ in a fully deterministic imaginary-time propagation for the ^1^Δ_*g*_ state of oxygen in an (8,6) active space.

**Figure 10:**
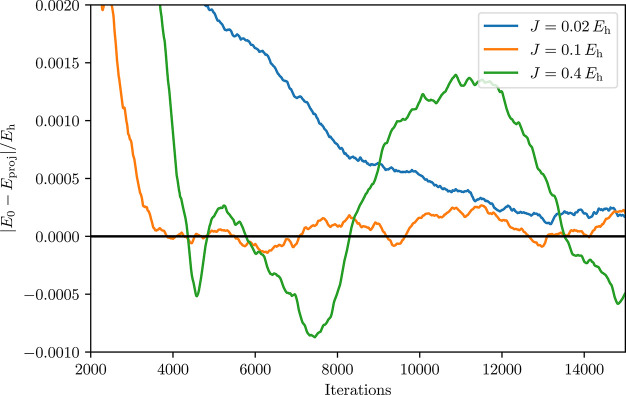
Moving average (window length is 200) of the projected energy of FCIQMC for different *J* values for the ^1^Δ_*g*_ state of oxygen in an (8,6) active space.

## 5. Appendix

In the following, we prove the existence of an optimal range of *J* values for the speed of convergence of the first-order spin penalty approach with the modified Hamiltonian

7

in FCIQMC and discuss strategies for selecting it. The derivation that follows applies to any other method that performs a stochastic or nonstochastic linearized imaginary-time propagation. We write the imaginary-time propagation of Ψ(τ = 0) as

8

without renormalization. After linearization we arrive at the projector

9

where *E*_0_ is the ground-state energy and the working equations arise from repeated application of *Ĝ* which is nothing else than the *power method* for the operator *Ĝ*. We will first discuss the speed of convergence of power methods and apply it to the linear projector *Ĝ*.

### 5.1 Power Method

The speed of convergence for the power method is given by the following theorem.^79^

**Theorem 1** (Speed of convergence power method). *Let**be a real, symmetric matrix with positive eigenvalues* λ_*i*_*sorted in decreasing order**and*

10

*so the largest eigenvalue λ*_1_*is non-degenerate. The eigenvectors corresponding to λ*_*i*_*are denoted with***u**_*i*_. *We denote the starting guess with***x**^(0)^*and define the guess-vector and guess-eigenvalue of the kth iteration as*

11

12

*If the starting guess has non-zero overlap with the eigenvector of highest eigenvalue* (⟨**x**^(0)^|**u**_1_⟩ ≠ 0) *then*eqs 11*and*12*converge to***u**_1_*and* λ_1_, *respectively. The convergence rates are given by*

13

*where θ*^(*k*)^ = *∠*(**x**^(*k*)^, **u**_1_).

For *J* > *J*_f,1_ the lowest eigenstate **u**_1_ is the same in all cases. Since we also use the same starting guess in all cases the deviation angle of the starting guess θ^(0)^ is the same everywhere and we will omit the sin^2^(θ^(0)^) term from further equations.

For further analysis we would like to emphasize that eq 13 is an *upper bound*, and depending on the energetically higher eigenvalues, λ_*i*_, *i* > 2, differences in convergence behavior may further occur.

We would like to showcase this with the three diagonal matrices from :

14

and a starting vector

15

The λ_*i*_, 1 ≤ *i* ≤ 3 eigenvalues were not chosen randomly, but were calculated as the first three eigenvalues of the projector *Ĝ* in the (9,9) active space on the manganese cluster for different values of *J*. **A** corresponds to *J* = (*J*_f,1_ + *J*_f,2_)/2, **B** corresponds to *J* = *J*_f,2_ + 0.0001 *E*_h_, and **C** corresponds to . The matrices **A**, **B**, and **C** have the same eigenvectors and the same spread of λ_1_ – λ_4_ = 1. By the ratio of λ_2_/λ_1_ and theorem 1 it is expected that matrices **B** and **C** have the same convergence behavior and converge faster than **A**.

In Figure 8, we see that the convergence in the long-time limit is indeed described by Theorem 1 and is only depending on the ratio of λ_2_/λ_1_. Nevertheless in the beginning there is faster convergence for **C** because the third component is projected out faster, due to a smaller λ_3_. So even for a constant ratio λ_2_/λ_1_ it is still advantageous to spread the distance between λ_1_ and the other eigenvalues.

### 5.2 Power Method Bounds in FCIQMC

In order to apply Theorem 1 to *Ĝ* we need to assume

16

and that the ground-state is non-degenerate. The assumption from eq 16 is usually fulfilled in FCIQMC, since the reduction of stochastic noise often requires even smaller time steps. We denote the eigenvalues of *Ĝ* with λ_*i*_. By construction we have 1 = λ_1_, and by assumption (eq 16), we have λ_*n*_ ≥ 0 which gives in total 1 = λ_1_ > λ_2_ ≥··· λ_*n*_ ≥ 0. Note that the highest eigenvalue λ_1_ of *Ĝ* belongs to the state with the lowest eigenvalue of *H*.

We will write the eigenvalues of the modified Hamiltonian and resulting variables like the time step with a prime superscript, ′. To simplify the discussion herein, we assume deterministic imaginary-time propagation and that Δτ is chosen maximal

17

for its respective Hamiltonian and value of *J*. We also assume

18

for the original Hamiltonian *Ĥ*.

#### 5.2.1 Case: *J*_f,1_ < *J* < *J*_f,2_

The ordering of eigenvalues of *Ĥ*^′^ is given by

19

We want to determine the relevant eigenvalues of

20

By construction we have

21

with Δ*E*_hs_ = *E*_hs,0_ – *E*_ls,0_ and Δ⟨*Ŝ*⟩ being the difference of *Ŝ* expectation value between the high- and low-spin state. Because of the assumption for Δτ (eq 17) we get

22

In total, we obtain (λ_1_ – λ_*n*_) = 1 and . As discussed in the main text we assume that the time step is not yet affected by *J*, so Δτ′ ≈ Δτ. We conclude that the convergence is bounded as

23

which gets tighter with increasing *J*.

#### 5.2.2 Case: *J*_f,2_ < *J* < *J*_f,τ_

The ordering of eigenvalues of *Ĥ*^′^ is given by

24

Since the energetic spacing between eigenstates of same spin is not affected by *J*·*Ŝ*^2^ we get

25

with Δ*E*_ls_ = (*E*_ls,1_ – *E*_ls,0_).

As in the previous case and by definition of *J*_f,τ_ we assume that the time step is not yet affected by *J*, so Δτ′ ≈ Δτ. As before we conclude that the convergence is bounded as

26

which is independent of *J*. Note that the other eigenvalues of *Ĝ* like λ_3_ are still affected by *J*, and the convergence is still slightly improved by increasing *J*.

#### 5.2.3 Case: *J* > *J*_f,τ_

A tilde will be used for this case. The ordering of the eigenvalues is given by

27

The energetic spacing between the two lowest states is independent of *J* and we get:

28

Again, we assume a maximum choice for the time step, so

29

As before, we conclude

30

For very large *J*, the spread of eigenvalues is dominated by the spacing between the spin eigenvalues, so we get

31

where Δ⟨*Ŝ*⟩_max_ is the difference between the lowest and highest possible spin state for that system. We get

32

This means that the convergence is bounded as

33

which gets tighter with decreasing *J*.

### 5.3 Analyzing All Cases Together

We summarize that the convergence improves with increasing *J* for *J*_f,1_ < *J* < *J*_f,2_. For *J* > *J*_f,τ_, the convergence deteriorates with increasing *J*. Between *J*_f,2_ and *J*_f,τ_, there is a plateau of near-optimal *J* values.

If we take the derivative of the two bounds (1 – Δτ(Δ*E*_hs_ + *J*Δ⟨*Ŝ*⟩)) and  after *J* we can see that in the case of *J* > *J*_f,τ_ the rate of change is smaller. This means that when in doubt it is better to choose *J* > *J*_f,τ_ than to choose *J* <*J*_f,2_; thus, it is better to “overshoot”.

It might be tempting to equate the two upper bounds from the separate cases to determine a unique *J*_opt_. This is not a defined operation because both expressions were separately derived under the assumption of either high or low *J*. In the scope of one of the bounds, the other one is not valid.

### 5.4 Numerical Tests for *J* Choice

In order to numerically confirm our derivations for the choice of optimal *J* values and to stay as close as possible to the assumptions in our previous derivation, we also performed a fully deterministic imaginary-time evolution for the systems that were discussed in our main text. The fully deterministic propagation was achieved by defining a semi-stochastic space for FCIQMC that ranged over the whole Hilbert space. The spacing of eigenvalues for the calculation of Δτ (eq 17) was estimated from the spread of diagonal values of the Hamiltonian matrix.

In Figure 9, we see the number of iterations required to achieve convergence up to 1 × 10^–5^*E*_h_ in a deterministic imaginary-time propagation for the ^1^Δ_*g*_ state of oxygen in the minimal (8,6) active space. If we keep the doubly logarithmic scale in mind, we see the previous derivations numerically confirmed. For *J*_f,1_ < *J* < *J*_f,2_, the convergence improves drastically with increasing *J*. For *J*_f,2_ < *J* < *J*_f,τ_, a plateau forms, but it is still better to choose *J* closer to *J*_f,τ_. For *J* > *J*_f,τ_ the convergence deteriorates with increasing *J*.

For a stochastic imaginary-time propagation the criterion of convergence up to 1 × 10^–5^*E*_h_ cannot be easily evaluated. Instead the moving average of the projected energy for three different *J* values is drawn in Figure 10. For this system, we had *J*_f,2_ = 0.03 *E*_h_ and *J*_f,τ_ ≈ 0.07 *E*_h_. The blue line describes a dynamic with *J* < *J*_f,2_, and the convergence improves considerably when going to higher *J* values as in the orange line. For values considerably above *J*_f,τ_ (green line), the quality of the calculation decreases again.
